# Blonanserin transdermal patch for treating delirium: A case series

**DOI:** 10.1002/pcn5.70341

**Published:** 2026-05-11

**Authors:** Mari Hemmi, Takuma Yamaguchi, Yuki Shigetsura, Daiki Hira, Hitoshi Tanimukai, Hirotsugu Kawashima, Yukiko Miyoshi, Hiroki Endo, Shunsaku Nakagawa, Tomohiro Terada

**Affiliations:** ^1^ Department of Clinical Pharmacology and Therapeutics Kyoto University Hospital Kyoto Japan; ^2^ Department of Psychiatric and Mental Health Nursing, Graduate School of Nursing Nagoya City University Nagoya Japan; ^3^ Department of Psychiatry, Graduate School of Medicine Kyoto University Kyoto Japan

**Keywords:** blonanserin, delirium, effectiveness, safety, transdermal patch

## Abstract

**Aim:**

Antipsychotics are commonly recommended for the treatment of delirium; however, alternative options are warranted due to the limitations of oral and injectable formulations. The blonanserin (BNS) transdermal patch may improve treatment adherence; however, evidence regarding its efficacy in managing delirium remains limited.

**Methods:**

This retrospective case series describes 51 cases of delirium managed with BNS patches at Kyoto University Hospital between January 2020 and June 2022. The effectiveness of the BNS patch for delirium, the rationale for its selection, and the associated adverse events were retrospectively evaluated using electronic medical records. Delirium was diagnosed according to the Diagnostic and Statistical Manual of Mental Disorders, 5th edition criteria by physicians from the consultation‐liaison or palliative care teams, and the symptoms were regularly monitored. Effectiveness was assessed based on non‐standardized clinical judgment documented in the medical records.

**Results:**

Hyperactive and mixed delirium were observed in 41.2% and 56.9% of patients, respectively. Overall, the therapeutic response rate across all patients was 84.3%. The main reasons for the selection of BNS patches were difficulty or inability to take oral medications (60.8%). Adverse events occurred in 29.4% of patients; all resolved after discontinuation of the BNS patch, and no serious or irreversible reactions were observed.

**Conclusion:**

These findings indicate that the BNS patch has potential as an effective treatment option for delirium, particularly in patients with challenges in the administration of medication. However, given the retrospective and exploratory nature of this study, the findings should be interpreted with caution.

## Graphical abstract

In a retrospective case series of 51 patients with delirium treated at a single center between 2020 and 2022, clinical improvement was observed in 84.3% of patients following the use of a blonanserin transdermal patch, often within 1–2 weeks. The patch was mainly selected for patients who had difficulty taking oral medications. Adverse events occurred in 29.4% of patients and were generally reversible. These findings suggest that the blonanserin transdermal patch may be a potential treatment option for delirium, although the results should be interpreted with caution because of the retrospective design.

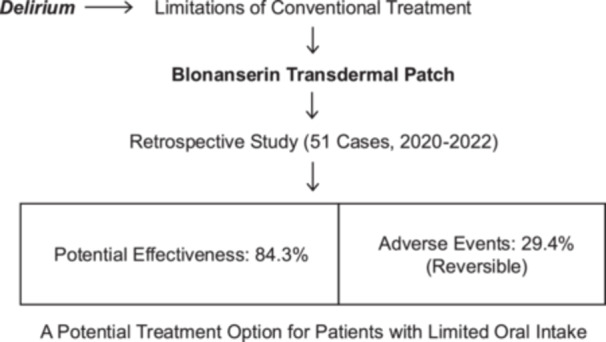

## INTRODUCTION

Delirium is an acute disorder of consciousness that occurs frequently in clinical settings,^1^, ^2^ characterized by cognitive dysfunction, disorientation, hallucinations, and delusions. This condition often leads to psychological distress for patients' family members,^3^ prolonged hospitalization,^4^ and increased medical expenses.^5^ Furthermore, delirium increases the risk of mortality and dementia,^6^ underscoring the importance of early treatment.

Most clinical guidelines advocate the use of antipsychotics, such as haloperidol, olanzapine, and risperidone, in patients with severe agitation or distress who present a substantial risk of harm to themselves or others.^7^, ^8^, ^9^, ^10^, ^11^, ^12^ Although antipsychotics can be administered orally or via injection, patients with delirium often have difficulty taking oral medications and rely on injectable drugs, such as haloperidol. However, typical antipsychotics, such as haloperidol, are associated with a higher incidence of adverse effects^13^ and, in older patients with dementia, an increased risk of mortality^14^, ^15^ compared to atypical antipsychotics. Furthermore, patients with delirium face a higher risk of self‐removal of the intravascular tube,^16^ complicating the use of injectable drugs. Consequently, alternative therapeutic approaches are required.

In 2019, the blonanserin (BNS) transdermal patch was approved as the first antipsychotic transdermal patch in Japan. Rivastigmine, the first transdermal patch for central nervous system diseases in Japan, is employed in the treatment of Alzheimer's disease and is reportedly user‐friendly for patients with limited disease awareness and adherence, as well as convenient for caregivers.^17^ Transdermal patches are an important treatment strategy for enhancing patient adherence to treatment. However, data on the effectiveness of BNS patches for treating delirium remain limited, despite evidence supporting the effectiveness of oral BNS in delirium.^18^ This study investigated the effectiveness and safety of BNS patches for the treatment of delirium at Kyoto University Hospital.

## MATERIALS AND METHODS

### Study samples

**Figure 1:**
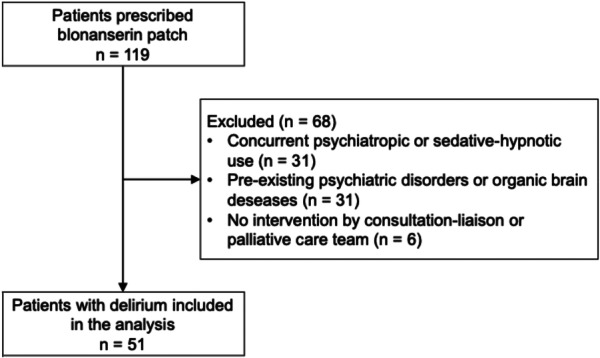
Flow diagram of patient selection.

We retrospectively reviewed the electronic medical records of inpatients at Kyoto University Hospital from January 2020 to June 2022. During this period, 119 patients were prescribed BNS patches. Patients with suspected delirium were referred to and assessed by psychiatrists from either the consultation‐liaison or palliative care teams, in accordance with the criteria outlined in the Diagnostic and Statistical Manual of Mental Disorders, 5th edition. These psychiatrists also participated in decisions regarding the application of the BNS patch. In addition, patient conditions and diagnoses were regularly reviewed and discussed in case conferences involving multiple psychiatrists, which supported the diagnostic consistency of delirium in this study. Of these, we excluded patients who had initiated other psychotropics or sedative‐hypnotic medications concurrently with the BNS patch (*n* = 31), those with pre‐existing psychiatric disorders or organic brain diseases (*n* = 31), and those who did not receive interventions from either the liaison or palliative care team (*n* = 6). Patients with pre‐existing psychiatric disorders or organic brain diseases were excluded to minimize confounding effects on delirium assessment, as these conditions can independently influence consciousness, behavior, and treatment response, making it difficult to evaluate delirium‐related symptom changes attributable to the intervention. Following the application of these exclusion criteria, 51 patients diagnosed with delirium and prescribed BNS patches were included in this analysis (Figure 1).

### Patient demographics

Data on age, sex, dementia status, medical history of delirium, cause of delirium, delirium symptoms, and psychotropic usage were collected from the patients' medical records. The psychotropics included antipsychotics, antidepressants, hypnotics, antiepileptic drugs, sedatives, anti‐dementia medications, narcotics, and herbal medicines (sansoninto and yokukansan) for the treatment of insomnia and neuropsychiatric disorders. Data were collected both prior to the onset of delirium and during the period between the onset of delirium and initiation of BNS patch treatment. Additionally, the reasons for selecting a BNS patch and the prescribed dosage were examined.

### Diagnosis of delirium and effectiveness of BNS patches

Delirium onset was diagnosed by a psychiatrist from the liaison or palliative care team based on the Diagnostic and Statistical Manual of Mental Disorders, 5th edition criteria. At Kyoto University Hospital, patients with psychiatric symptoms, such as agitation and irritability, were consistently monitored and documented in their medical records by psychiatrists or nurses from the liaison or palliative care team. In this study, the effectiveness of BNS patches for delirium was evaluated from the start to the end of the patch application using medical records documented by physicians and nurses. A treatment response was defined as a clinically meaningful improvement in delirium‐related symptoms documented in the medical records, such as reductions in restlessness, agitation, hallucinations, or dangerous behaviors. Improvement was determined based on physicians' and nurses' assessments of behavioral stabilization and decreased need for pharmacological or physical intervention.

Data extraction from the electronic medical records and data analysis were conducted by independent individuals.

### Adverse events

We surveyed adverse events that occurred after the initiation of the BNS patch, focusing on sedation, somnolence, extrapyramidal symptoms, and corrected QT interval (QTc) prolongation, which are potential side effects of BNS patches. We also investigated the incidence of BNS patch discontinuation due to adverse events.

## RESULTS

### Patient characteristics

Tables 1 and S1 show the characteristics of the 51 patients included in this study. Among the patients, hyperactive delirium was observed in 41.2% and mixed delirium in 56.9%. The most common symptoms were emotional agitation (37.3%) and psychomotor hyperactivity (35.3%), followed by hallucination and violent behavior. The most frequently prescribed psychotropics before the onset of delirium were hypnotics, such as ramelteon (21.6%) and lemborexant (15.7%), which were used to regulate the sleep–wake cycle during hospitalization. After the onset of delirium, the same hypnotic agents were also prescribed, with lemborexant (23.5%) and ramelteon (15.7%) being commonly used. Other psychotropics used for delirium were antipsychotics such as risperidone (39.2%), haloperidol (31.4%) and quetiapine (13.7%). The detailed clinical characteristics of the 51 patients are provided in Table S2.

**Table 1:** Patient demographics and clinical characteristics.

		Patients using blonanserin transdermal patches *n* = 51
Sex, male/female, *n*		34/17
Age, years, median (range)		79 (51–95)
History of dementia, *n* (%)		8 (15.7)
Presumed cause of delirium, *n* (%)	Drug‐related	12 (23.5)
	Infection	13 (25.5)
	Heart failure	6 (11.8)
	Cerebral infarction	3 (5.9)
	Post‐surgery	3 (5.9)
	Chemotherapy	2 (3.9)
	Anemia	1 (2.0)
	Dehydration	1 (2.0)
	Hypercalcemia	1 (2.0)
	Hypernatremia	1 (2.0)
Type of delirium, *n* (%)	Hyperactive	21 (41.2)
	Mixed	29 (56.9)
	Hypoactive	1 (2.0)
Predominant symptoms of delirium, *n* (%)	Emotional agitation	19 (37.3)
	Psychomotor hyperactivity	18 (35.3)
	Hallucination	17 (33.3)
	Violent behavior	17 (33.3)
	Irritability	14 (27.5)
	Insomnia	13 (25.5)
	Confusion of thought	7 (13.7)
	Agitation	2 (3.9)
	Depression	1 (2.0)
Psychotropic drugs before developing delirium, *n* (%)	None	15 (29.4)
	Benzodiazepine receptor agonists	9 (17.6)
	Ramelteon	11 (21.6)
	Lemborexant	8 (15.7)
	Suvorexant	3 (5.9)
	Quetiapine	3 (5.9)
	Haloperidol	3 (5.9)
	Other antipsychotics	4 (7.8)
	Antidepressants	8 (15.7)
	Antiepileptic drugs	5 (9.8)
	Anti‐dementia drugs	3 (5.9)
	Others	11 (21.6)
Psychotropic drugs for delirium before using blonanserin transdermal patch, *n* (%)	None	13 (25.5)
	Benzodiazepine receptor agonist	2 (3.9)
	Ramelteon	8 (15.7)
	Lemborexant	12 (23.5)
	Suvorexant	2 (3.9)
	Antipsychotics	25 (49.0)
	Haloperidol	16 (31.4)
	Quetiapine	7 (13.7)
	Risperidone	20 (39.2)
	Other antipsychotics	5 (9.8)
	Antidepressants	10 (19.6)
	Others	17 (33.3)

Table 2 presents the treatment durations and reasons for selecting BNS patches. Twenty‐two (43.1%) patients received a 20 mg BNS patch. The median BNS patch dose was 40 mg/day (range, 20–80 mg/day). The median time until observed effectiveness was 6 days (range, 1–23 days; interquartile range, 3.5–8.5 days). Improvement was observed within 1–6 days in 25 patients (49.0%), within 7–13 days in 15 patients (29.4%), and ≥14 days in 3 patients (5.9%). Among patients who showed a positive response (*n* = 43), the corresponding proportions were 58.1%, 34.9%, and 7.0%, respectively. In addition, the median duration of BNS patch use was 10 days (range, 2–35 days; interquartile range, 7–13 days). The most common duration of use was 7–13 days, observed in 25 (49.0%) patients. The main reasons for choosing BNS patches were the difficulty or inability to take oral medications in 31 (60.8%) patients.

**Table 2:** Treatment duration and reason for selection of blonanserin transdermal patches.

	Patients using blonanserin transdermal patches *n* = 51
Time until observed effectiveness (days), *n* (%) [%]	
Less than 7 days	25 (49.0) [58.1]
More than 7 days and less than 14 days	15 (29.4) [34.9]
More than 14 days	3 (5.9) [7.0]
Period of use (days), *n* (%)	
Less than 7 days	12 (23.5)
More than 7 days and less than 14 days	25 (49.0)
More than 14 days	14 (27.5)
Reason for selection, *n* (%)	
Difficulty or inability to take oral medications	31 (60.8)
Avoidance of oral medication (water restriction)	1 (2.0)
Inability to administer injection drugs	1 (2.0)
Insufficient efficacy with other drugs	13 (25.5)
Stable therapeutic effect throughout the day	3 (5.9)
Dangerous behavior	2 (3.9)

*Note*: Percentages in parentheses are based on the total study population (*n* = 51). Values in square brackets indicate percentages among patients who showed a positive response (*n* = 43).

### Effectiveness of BNS patches for delirium

In this study, 43 patients (84.3%) responded positively to the BNS patch for delirium, with the majority of patients showing improvement in their symptoms (Table 3). However, eight patients did not show a positive response. In several patients, metabolic abnormalities such as electrolyte disturbances or anemia were considered potential contributing factors to delirium onset. Specifically, hypernatremia, hypercalcemia, dehydration, and anemia were identified as possible etiological factors. In all cases, these abnormalities improved following appropriate medical management, including fluid therapy or blood transfusion. However, the timing of correction did not consistently coincide with improvement in delirium symptoms, suggesting that these factors alone did not fully account for the clinical course of delirium in these patients.

**Table 3:** Effect of blonanserin transdermal patches on the type and symptoms of delirium.

	Effective, *n* (%)	Ineffective, *n* (%)
All patients (*n* = 51)	43 (84.3)	8 (15.7)
Type of delirium		
Hyperactive delirium (*n* = 21)	17 (81.0)	4 (19.0)
Mixed delirium (*n* = 29)	25 (86.2)	4 (13.8)
Hypoactive delirium (*n* = 1)	1 (100)	0 (0)
Symptoms of delirium		
Emotional agitation (*n* = 19)	16 (84.2)	3 (15.8)
Psychomotor hyperactivity (*n* = 18)	17 (94.4)	1 (5.6)
Hallucination (*n* = 17)	16 (94.1)	1 (5.9)
Violent behavior (*n* = 17)	16 (94.1)	1 (5.9)
Irritability (*n* = 14)	10 (71.4)	4 (28.6)
Insomnia (*n* = 13)	12 (92.3)	1 (7.7)
Confusion of thought (*n* = 7)	4 (57.1)	3 (42.9)
Agitation (*n* = 2)	2 (100)	0 (0)
Depression (*n* = 1)	1 (100)	0 (0)

**Figure 2:**
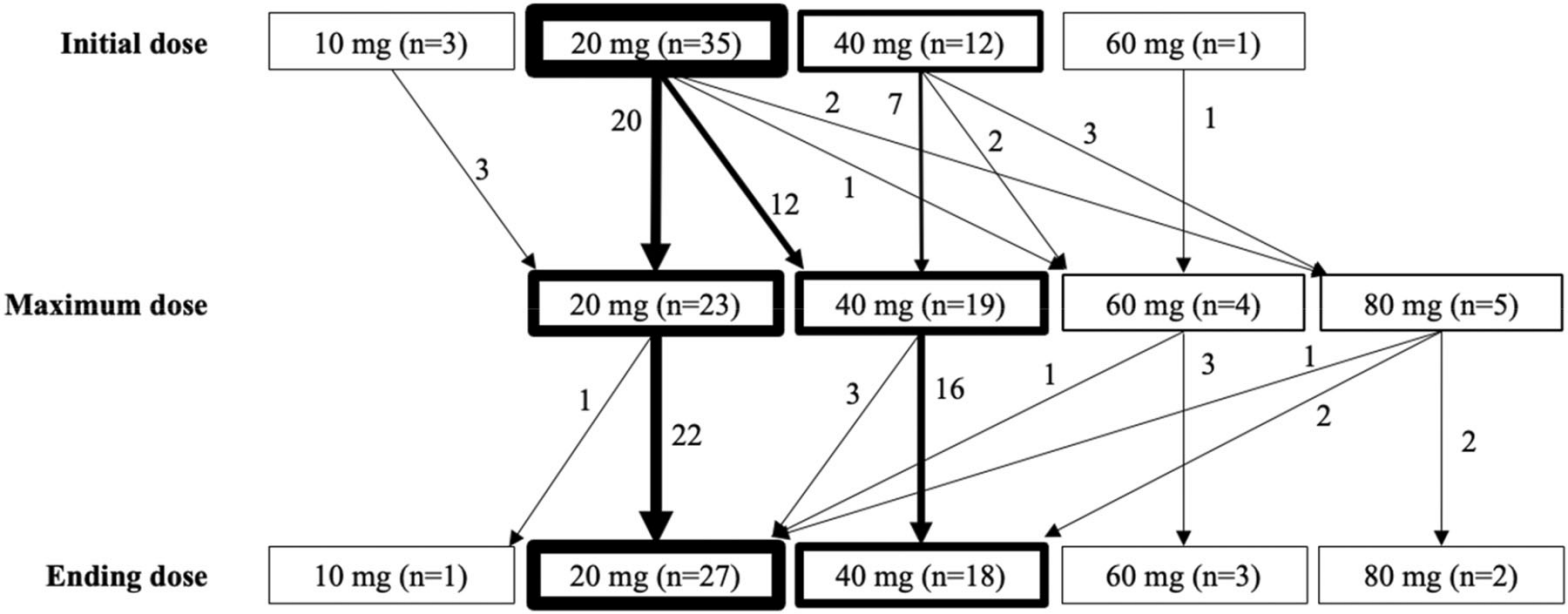
Initial, maximum, and final doses of blonanserin transdermal patches in patients with delirium (*n* = 51). Dose changes between 10, 20, 40, 60, and 80 mg/day are illustrated using arrows. The thicknesses of the lines and arrows correspond to the number of individuals, with thicker arrows indicating larger numbers.

Figure 2 presents the number of patients treated with the BNS patch at the starting, maximum, and final doses, with 20 mg being the most common dose (starting dose: 35 patients, 68.6%; maximum dose: 23 patients, 45.1%; and final dose: 27 patients, 52.9%).

### Adverse events

Table 4 shows the adverse events and reasons for discontinuation. Of the 51 patients, 15 (29.4%) experienced adverse events. The incidence of adverse events was comparable between patients using the 20 mg BNS patch (36.8%; 7 out of 19 patients) and those using 40 mg or higher (32.1%; 9 out of 28 patients). A patient with skin disorders had pre‐existing skin issues that worsened by the BNS patch.

**Table 4:** Adverse events and reason for discontinuation of blonanserin transdermal patches.

	Patients using blonanserin transdermal patches *n* = 51
Adverse events, *n* (%)	15 (29.4)
Sedation	4 (7.8)
Somnolence	5 (9.8)
Extrapyramidal symptoms	6 (11.8)
Increase of CK	1 (2.0)
Skin disorder	1 (2.0)
Reason for the BNS patch cessation, *n* (%)	
Adverse events	8 (15.7)
Sedation	2
Somnolence	1
Extrapyramidal symptoms	5
Skin disorder	1
Resumption of oral medication	1 (2.0)
Recover from delirium	16 (31.4)
Discharge and transfer	22 (43.1)
Death	7 (13.7)

Abbreviations: BNS, blonanserin; CK, creatine kinase.

The most common reasons for discontinuation of BNS patches were discharge or hospital transfer (43.1%) and improvement in delirium (31.4%). Among the reasons for discontinuation, seven patients died during the observation period; however, these deaths were attributed to the progression of pre‐existing medical conditions and were not considered related to the use of the BNS patch. Because some patients had more than one reason for BNS patch cessation, the categories of discontinuation are not mutually exclusive. In particular, three patients discontinued the BNS patch due to adverse events after experiencing improvement in delirium. Adverse events led to discontinuation in eight patients (16%), with five patients experiencing extrapyramidal symptoms. Extrapyramidal symptoms included dystonia, gait disturbance, dysphagia, and sialorrhea, typically occurring within 1–5 days after initiation of the BNS patch. Most cases were mild, and all symptoms improved after dose reduction or discontinuation, with no persistent deficits observed. Overall, adverse events were reversible and resolved with appropriate management, including dose reduction or discontinuation of the BNS patch.

Electrocardiographic monitoring after BNS initiation was performed in six patients at the discretion of the treating physicians, and no clinically significant QTc prolongation or arrhythmic events were documented. However, QTc‐related adverse events may have been under‐ascertained because ECG monitoring was not systematically performed.

One patient experienced an increase in creatine kinase levels (from 16 to 544 U/L on Day 2 after BNS patch initiation), which decreased after discontinuation of the BNS patch without further clinical complications.

Overall, adverse events were reversible and resolved with appropriate management, including dose reduction or discontinuation of the BNS patch.

## DISCUSSION

Delirium, an acute syndrome characterized by disturbances in attention, awareness, and cognition, requires prompt treatment.^2^ However, the symptoms of delirium can hinder the administration of oral and injectable medication, resulting in treatment delays associated with increased risks of mortality.^19^ Although an asenapine transdermal patch was approved and marketed in the United States in 2020, the BNS patch is currently the only transdermal antipsychotic formulation available in Japan. Therefore, the BNS patch may be considered for recommendation in cases of delirium where other treatment options are unsuitable. In this study, BNS patches achieved a therapeutic response rate of 84%, demonstrating their substantial effectiveness in the management of delirium. Although a previous study reported a response rate of 97% for oral BNS,^18^ differences in study design, patient characteristics, and outcome assessment methods should be considered when interpreting the present results. In particular, most patients in the present study had already received other psychotropic treatments and experienced difficulties with medication administration, which may have influenced treatment response. In addition, because effectiveness was assessed based on clinical judgment documented in medical records, variability among clinicians, differences between consultation‐liaison and palliative care services, and heterogeneity in documentation practices may have influenced outcome classification, which should be considered when interpreting the results. These findings may indicate that BNS patches provide a clinically meaningful treatment option for patients with limited access to or tolerance for conventional therapies. Overall, our findings align with a previous report,^20^ supporting the clinical utility of BNS patches in managing delirium. While the previous study primarily focused on the acceptability of BNS patches and their role in preventing the recurrence of delirium in patients who refused oral medication, the present study differs in that it evaluated the effectiveness and safety of BNS patches in a broader real‐world clinical population, including patients with various indications for transdermal therapy beyond medication refusal.

Recently, interest has grown in non‐oral formulations, with reports suggesting their usefulness in reducing postoperative delirium.^21^ Such developments indicate a clinical demand for alternatives to oral or injectable therapies. In this context, the transdermal patch offers several advantages, including ease of use, reduced dosing frequency, and low invasiveness, while also potentially reducing the burden on medical staff and caregivers. In the present study, difficulty with oral medication was the primary reason for switching to BNS patches. Therefore, the transdermal route may represent a less invasive option for patients who are unable or unwilling to take oral or injectable formulations, while also improving overall treatment tolerability compared with conventional approaches.^22^

In the treatment of delirium, medication‐related adverse effects can complicate the clinical assessment of patients. BNS demonstrates a strong affinity for D_2_ receptors and a low affinity for H_1_, α_1,_ and M_1_ receptors.^23^, ^24^ It possesses a favorable safety profile, with fewer anticholinergic or cardiovascular adverse effects. Across this study population, adverse events associated with the BNS patch were generally manageable and reversible. Although sedation, somnolence, and extrapyramidal symptoms were observed, all events improved after dose reduction or discontinuation, and no serious or irreversible adverse events were identified. Therefore, it serves as a valuable treatment for delirium accompanied by various comorbidities. In this study, adverse events requiring discontinuation occurred in 16% of patients; however, these events were generally manageable and reversible. Importantly, because the use of the BNS patch for delirium is off‐label, clinicians may have adopted a more cautious approach, leading to earlier discontinuation than would typically occur in standard practice. This observed rate of discontinuation may reflect careful monitoring rather than an inherent safety concern. Skin reactions are an important consideration for transdermal formulations. In this study, skin‐related adverse events were infrequent and occurred in a single patient with pre‐existing skin conditions, in whom symptoms improved after discontinuation of the BNS patch. Although the incidence was low, careful monitoring of the application site is warranted, particularly in patients with underlying dermatological vulnerability. Notably, the transdermal patch formulation offers the additional advantages of maintaining consistent plasma concentration^25^ and allowing for immediate cessation of treatment by removing the patch when adverse reactions occur. These characteristics hold significant clinical relevance in the context of delirium, where the rapid modification of therapeutic interventions is frequently necessary. Consequently, our findings endorse the BNS patch as a viable and safe option for the management of delirium.

This retrospective study had some limitations. First, this study was a retrospective, single‐center study with a relatively small sample size and no control group, limiting the robustness of the evidence. Moreover, the use of BNS patches for delirium is off‐label, which restricts their application to specific cases and may introduce a selection bias. In particular, BNS patches were more likely to be prescribed to patients with prominent hyperactive or mixed symptoms requiring active pharmacological intervention. In contrast, hypoactive delirium is often under‐recognized in routine clinical practice, which may partly explain its low representation in this cohort and limit the generalizability of the findings to that subtype. In addition, hypoactive delirium may be less likely to prompt pharmacological intervention in routine clinical practice, as patients typically exhibit fewer disruptive or dangerous behaviors; consequently, non‐pharmacological management or careful observation may be prioritized, which could have further contributed to the low number of such cases treated with the BNS patch. Second, the evaluation of effectiveness was based on clinical judgment, and no validated delirium assessment scales, such as the Confusion Assessment Method for the Intensive Care Unit (CAM‐ICU), Delirium Rating Scale‐Revised‐98 (DRS‐R‐98), or Memorial Delirium Assessment Scale (MDAS), were used in this study. This approach inevitably introduces subjectivity and potential observer bias, as clinical judgments may vary among clinicians. In addition, the symptoms of delirium can naturally fluctuate and often improve as the underlying condition resolves, complicating the isolation of the direct effects of BNS patches. Moreover, symptom improvement may have been influenced by concurrent medical treatments and the delayed effects of psychotropic medications administered prior to BNS patch initiation, representing potential confounding factors. Furthermore, the timing of evaluation varied among patients, as the patch was often initiated after other treatments had been attempted, and the duration of application differed across cases. Importantly, although the treatment duration frequently exceeded one week, clinical improvement was observed within a relatively short period in most patients. Among patients who showed a positive response, approximately 58% demonstrated improvement within the first week, and over 90% within one to two weeks after initiation of the BNS patch. These findings should be interpreted in the clinical context, as BNS patches were often introduced after prior psychotropic treatments or in patients with difficulty in oral administration, representing relatively complex clinical situations. In addition, compared with oral antipsychotics, the transdermal formulation may be associated with a more gradual onset of clinical effect. However, as suggested in previous studies,^20^ even such a response profile may have clinical value in patients who have difficulty with oral administration or adherence. Therefore, the observed treatment duration likely reflects real‐world treatment sequences rather than a delay in therapeutic effect. Finally, the study included patients from various clinical departments with diverse comorbidities, potentially introducing heterogeneity in both the etiology and treatment response. In addition, electrocardiographic QTc monitoring was not performed in a standardized manner and was conducted at the discretion of the treating physicians, which may have led to underestimation of QTc‐related adverse events. Therefore, the findings should be interpreted as descriptive clinical impressions rather than definitive evidence of efficacy.

## CONCLUSION

BNS patches were associated with improvement in delirium symptoms and were generally well tolerated in this study. However, given the exploratory nature of the analysis, these findings should be interpreted cautiously. Although careful patient selection is required, BNS patches may represent a potentially useful treatment option for delirium, particularly in patients who have difficulty with medication administration.

## AUTHOR CONTRIBUTIONS

Mari Hemmi and Takuma Yamaguchi contributed equally to this study. Mari Hemmi, Takuma Yamaguchi, and Yuki Shigetsura conceived and designed the study. Mari Hemmi, Takuma Yamaguchi, and Yuki Shigetsura drafted the manuscript, obtained the patient data, and analyzed the data. Hitoshi Tanimukai and Hirotsugu Kawashima provided feedback in psychiatry. Daiki Hira, Hitoshi Tanimukai, Hirotsugu Kawashima, Yukiko Miyoshi, Hiroki Endo, Shunsaku Nakagawa, and Tomohiro Terada critically reviewed the manuscript. All authors participated in drafting the discussion of the results and reviewed the manuscript.

## CONFLICT OF INTEREST STATEMENT

The authors declare no conflicts of interest.

## ETHICS APPROVAL STATEMENT

This study was performed in accordance with the “Ethical Guidelines for Life Science and Medical Research Involving Human Subjects” and was reviewed by the Ethics Committee of the Kyoto University Graduate School of Medicine, School of Medicine, and Faculty of Medicine Hospital (approval number: R0545‐2). All study participants provided opt‐out consent because of the retrospective nature of the study.

Separately from this research approval, the clinical use of the BNS patch for delirium was conducted as an off‐label treatment in routine care. Because this indication is not formally approved, its clinical use was reviewed and approved by the Off‐Label Drug Evaluation Working Group under the Pharmaceutical Safety Management Subcommittee, which evaluates the appropriateness of off‐label medication use in individual clinical settings.

Within this framework, the administration of the BNS patch was determined by clinical necessity on an individual basis, involving psychiatrists from either the consultation‐liaison or palliative care teams. Consequently, this study was structured as a retrospective observational study utilizing routinely collected clinical data, with no interventions assigned for research purposes.

Prior to administration of the BNS patch, patients and their families were provided with an explanation regarding the off‐label nature of the treatment, as well as its potential benefits and risks, and verbal consent was obtained as part of clinical practice. In accordance with routine clinical practice for off‐label medication use at our institution, written consent or formal documentation in the medical records was not routinely required; however, explanations were provided to all patients included in this study.

## PATIENT CONSENT STATEMENT

All study participants provided opt‐out consent because of the retrospective nature of the study.

## CLINICAL TRIAL REGISTRATION

N/A.

## Supporting information


Supporting File 1

Supporting File 2

## Data Availability

The data that support the findings of this study are available in the supplementary material of this article.
