# Supplementary material for: Blonanserin transdermal patch for treating delirium: A case series
Source: PCN Rep. 2026 May 11;5(2):e70341. doi: 10.1002/pcn5.70341 (PMC13158812; doi:10.1002/pcn5.70341)
Supplement: Supplementary file 1 — Supporting File 1 [file PCN5-5-e70341-s002.docx]

Table S1. Patient demographics and clinical characteristics

|  |  | Patients using blonanserin transdermal patches n = 51 |
| --- | --- | --- |
| Sex, male/female, n | | 34 / 17 |
| Age, years, median (range) | | 79 (51–95) |
| History of dementia, n (%) | | 8 (15.7) |
| Presumed cause of delirium, n (%) | Drug-related | 12 (23.5) |
|  | Steroid | 7 |
|  | Fentanyl | 4 |
|  | Hydromorphone | 1 |
|  | Infection | 13 (25.5) |
|  | Pneumonia | 7 |
|  | Pyothorax | 1 |
|  | Meningoencephalitis | 1 |
|  | Bacteremia | 1 |
|  | Urinary tract infection | 1 |
|  | Meningitis | 1 |
|  | Cystitis | 1 |
|  | Heart failure | 6 (11.8) |
|  | Cerebral infarction | 3 (5.9) |
|  | Post-surgery | 3 (5.9) |
|  | Stent graft infection | 1 |
|  | Posterior thoracic decompression fixation | 1 |
|  | Arteriosclerosis obliterans | 1 |
|  | Chemotherapy | 2 (3.9) |
|  | Anemia | 1 (2.0) |
|  | Aortic dissection | 1 (2.0) |
|  | Cardiogenic cerebral embolism | 1 (2.0) |
|  | Chronic subdural hematoma | 1 (2.0) |
|  | Dehydration | 1 (2.0) |
|  | Diabetic ketoacidosis | 1 (2.0) |
|  | Distress due to breathing difficulties | 1 (2.0) |
|  | Hepatic encephalopathy | 1 (2.0) |
|  | Hypercalcemia | 1 (2.0) |
|  | Hypernatremia | 1 (2.0) |
|  | Inflammation | 1 (2.0) |
|  | Pleural effusion | 1 (2.0) |
| Type of delirium, n (%) | Hyperactive delirium | 21 (41.2) |
|  | Mixed delirium | 29 (56.9) |
|  | Hypoactive delirium | 1 (2.0) |
| Predominant symptoms of delirium, n (%) | Emotional excitement | 19 (37.3) |
|  | Psychomotor hyperactivity | 18 (35.3) |
|  | Hallucination | 17 (33.3) |
|  | Violent behavior | 17 (33.3) |
|  | Irritability | 14 (27.5) |
|  | Insomnia | 13 (25.5) |
|  | Confusion of thought | 7 (13.7) |
|  | Agitation | 2 (3.9) |
|  | Depression | 1 (2.0) |
| Psychotropic drugs before developing delirium, n (%) | None | 15 (29.4) |
|  | Benzodiazepine receptor agonists | 9 (17.6) |
|  | Brotizolam | 2 |
|  | Estazolam | 1 |
|  | Etizolam | 2 |
|  | Zolpidem | 2 |
|  | Eszopiclone | 1 |
|  | Zopiclone | 1 |
|  | Midazolam | 1 |
|  | Sleeping pills (excluding benzodiazepine receptor agonists) | 15 (29.4) |
|  | Ramelteon | 11 |
|  | Lemborexant | 8 |
|  | Suvorexant | 3 |
|  | Antipsychotics | 8 (15.7) |
|  | Quetiapine | 3 |
|  | Haloperidol | 3 |
|  | Perospirone | 1 |
|  | Prochlorperazine | 2 |
|  | Risperidone | 1 |
|  | Antidepressants | 8 (15.7) |
|  | Mirtazapine | 3 |
|  | Trazodone | 1 |
|  | Sertraline | 1 |
|  | Duloxetine | 1 |
|  | Antiepileptic drugs | 5 (9.8) |
|  | Levetiracetam | 2 |
|  | Lacosamide | 1 |
|  | Perampanel | 2 |
|  | Clonazepam | 2 |
|  | Carbamazepine | 1 |
|  | Phenytoin | 1 |
|  | Phenobarbital | 1 |
|  | Anti-dementia drugs | 3 (5.9) |
|  | Memantine | 2 |
|  | Rivastigmine | 1 |
|  | Hydroxyzine pamoate | 2 (3.9) |
|  | Amantadine | 1 (2.0) |
|  | Yokukansan | 4 (7.8) |
|  | Dexmedetomidine | 7 (13.7) |
|  | Propofol | 4 (7.8) |
| Psychotropic drugs for delirium before using blonanserin transdermal patch, n (%) | None | 13 (25.5) |
|  | Benzodiazepine receptor agonist | 2 (3.9) |
|  | Midazolam | 1 |
|  | Eszopiclone | 1 |
|  | Sleeping pills (excluding benzodiazepine receptor agonists) | 14 (27.5) |
|  | Ramelteon | 8 |
|  | Lemborexant | 12 |
|  | Suvorexant | 2 |
|  | Antipsychotics | 25 (49.0) |
|  | Haloperidol | 16 |
|  | Chlorpromazine | 1 |
|  | Quetiapine | 7 |
|  | Risperidone | 20 |
|  | Perospirone | 2 |
|  | Olanzapine | 2 |
|  | Asenapine | 1 |
|  | Antidepressants | 10 (19.6) |
|  | Trazodone | 4 |
|  | Mirtazapine | 1 |
|  | Mianserin | 1 |
|  | Tiapride | 1 (2.0) |
|  | Dexmedetomidine | 12 (23.5) |
|  | Hydroxyzine pamoate | 6 (11.8) |
|  | Propofol | 2 (3.9) |
|  | Sansoninto | 1 (2.0) |
