# Supplementary material for: Blonanserin transdermal patch for treating delirium: A case series
Source: PCN Rep. 2026 May 11;5(2):e70341. doi: 10.1002/pcn5.70341 (PMC13158812; doi:10.1002/pcn5.70341)
Supplement: Supplementary file 2 — Supporting File 2 [file PCN5-5-e70341-s001.docx]

Table S2. Detailed clinical characteristics of the 51 patients

| **Patient No.** | **Sex** | **Age (years)** | **History of dementia** | **Cause of delirium** | **Type of delirium** | **Symptoms of delirium** | **Psychotropic drugs before developing delirium** | **Psychotropic drugs for delirium before using BNS patch** | **Maximum dose of BNS patch (mg)** | **Time until observed effectiveness**  **(days)** | **Period of use (days)** | **Reason for selecting BNS patch** | **Treatment responsiveness** | **Documentation of evaluation by physicians and nurses in medical records** | **Adverse events** | **Reason for the end of follow-up or discontinuation of BNS patch** |
| --- | --- | --- | --- | --- | --- | --- | --- | --- | --- | --- | --- | --- | --- | --- | --- | --- |
| 1 | Male | 74 | None | Post-surgery  (Stent graft infection) | Hyperactive delirium | Emotional excitement  Hallucination | Lemborexant  Mirtazapine  Ramelteon  Zolpidem | Dexmedetomidine | 20 | 1 | 2 | Inability to take oral medications | Effective | Resolution of agitation and irritability | None | Became able to take oral medication |
| 2 | Male | 83 | Yes | Meningitis | Mixed delirium | Irritability  Violent behavior | Ramelteon | Dexmedetomidine  Haloperidol  Quetiapine | 20 | 4 | 9 | Rejection of oral medication | Effective | No agitation | None | Improvement of delirium |
| 3 | Male | 86 | Yes | Cardiogenic cerebral embolism | Hyperactive delirium | Emotional excitement  Irritability | Etizolam  Hydroxyzine pamoate | - | 20 | 2 | 8 | Rejection of oral medication | Effective | Improvement of delirium | None | Improvement of delirium |
| 4 | Male | 87 | None | Infection  (Urinary tract infection) | Mixed delirium | Hallucination  Insomnia  Violent behavior | - | Haloperidol  Lemborexant  Ramelteon | 40 | 7 | 7 | Rejection of oral medication | Effective | Improvement of delirium | Mild dysarthria (Extrapyramidal symptom)  Somnolence | Adverse event,  Improvement of delirium |
| 5 | Female | 77 | Yes | Anemia | Mixed delirium | Hallucination | - | Ramelteon  Risperidone  Suvorexant  Quetiapine | 20 | 5 | 6 | Inability to administer injection drugs  (Self-extraction of the intravascular tube) | Effective | Normal orientation | Mild gait disturbance (Extrapyramidal symptom) | Adverse event,  Improvement of delirium |
| 6 | Male | 87 | None | Infection  (Pneumonia) | Mixed delirium | Irritability  Violent behavior | Estazolam  Haloperidol | - | 20 | 3 | 12 | Inability to take oral medications | Effective | Good sleep control  No agitation | Sedation | Adverse event,  Improvement of delirium |
| 7 | Female | 91 | Yes | Cerebral infarction | Hyperactive delirium | Emotional excitement  Irritability  Violent behavior | Lemborexant  Memantine  Ramelteon  Rivastigmine  Yokukansan | - | 20 | 2 | 7 | Rejection of oral medication | Effective | Remaining calm | None | Discharge from hospital |
| 8 | Male | 67 | None | Hepatic encephalopathy | Hyperactive delirium | Emotional excitement  Irritability  Violent behavior | - | Haloperidol | 40 | 2 | 2 | Inability to take oral medications | Effective | Resolution of verbal and physical aggression | None | Discharge from hospital |
| 9 | Female | 61 | None | Chemotherapy  (Brain tumor) | Mixed delirium | Agitation  Emotional excitement  Irritability | Lacosamide  Lemborexant  Levetiracetam  Perampanel | Quetiapine | 40 | 6 | 7 | Inability to take oral medications | Effective | Remaining calm | None | Improvement of delirium |
| 10 | Male | 91 | None | Chronic subdural hematoma | Mixed delirium | Insomnia  Irritability  Violent behavior | Brotizolam  Quetiapine | Eszopiclone  Lemborexant  Ramelteon  Risperidone  Sansoninto | 40 | 5 | ≧14 | Insufficient efficacy with other drugs | Effective | Improvement of delirium | Skin disorder | Adverse event |
| 11 | Female | 84 | None | Use of steroid | Mixed delirium | Emotional excitement  Insomnia  Irritability | Mirtazapine  Ramelteon | Lemborexant  Perospirone  Trazodone | 20 | Ineffective | 6 | Insufficient efficacy with other drugs | Ineffective | Persistence of irritability and hypersensitivity | None | Transfer to another hospital |
| 12 | Female | 82 | None | Use of fentanyl | Mixed delirium | Emotional excitement  Psychomotor hyperactivity | Lemborexant | Asenapine  Dexmedetomidine  Haloperidol  Hydroxyzine pamoate  Quetiapine | 20 | 5 | ≧14 | Expectation of stable medicinal effect | Effective | Improvement of delirium | None | Discharge from hospital |
| 13 | Male | 75 | None | Infection  (Pyothorax) | Mixed delirium | Hallucination  Psychomotor hyperactivity | Ramelteon  Suvorexant | Haloperidol  Lemborexant  Quetiapine | 40 | Ineffective | 13 | Inability to take oral medications | Ineffective | Persistence of delirium symptoms | Sedation | Death |
| 14 | Mele | 72 | None | Use of fentanyl | Mixed delirium | Psychomotor hyperactivity | - | - | 20 | 7 | ≧14 | Inability to take oral medications | Effective | Improvement of delirium | None | Transfer to another hospital |
| 15 | Female | 95 | None | Infection  (Pneumonia) | Mixed delirium | Confusion of thought | Ramelteon | Dexmedetomidine  Haloperidol | 20 | Ineffective | 5 | Rejection of oral medication | Ineffective | Difficulty following commands | None | Transfer to another hospital |
| 16 | Male | 88 | Yes | Use of steroid | Mixed delirium | Confusion of thought  Emotional excitement | Memantine | - | 20 | 4 | 4 | Rejection of oral medication | Effective | No agitation | Sedation | Adverse event |
| 17 | Male | 67 | None | Infection  (Bacteremia) | Hyperactive delirium | Insomnia  Psychomotor hyperactivity | Dexmedetomidine  Midazolam  Zolpidem | Lemborexant  Ramelteon | 40 | 8 | ≧14 | Insufficient efficacy with other drugs | Effective | Good sleep control  Improvement of delirium | None | Improvement of delirium |
| 18 | Female | 75 | None | Infection  (Meningoencephalitis) | Hyperactive delirium | Confusion of thought  Irritability | Lemborexant, Yokukansan | Risperidone | 20 | 6 | 6 | Expectation of stable medicinal effect | Effective | Remaining calm | Elevation of creatine kinase  Extrapyramidal symptoms | Transfer to another hospital |
| 19 | Female | 85 | None | Cerebral infarction | Hyperactive delirium | Agitation  Confusion of thought  Psychomotor hyperactivity | - | Lemborexant  Ramelteon  Quetiapine | 20 | 10 | 10 | Insufficient efficacy with other drugs | Effective | Improvement of delirium | None | Transfer to another hospital |
| 20 | Female | 51 | None | Post-surgery  (Posterior thoracic decompression fixation) | Hyperactive delirium | Hallucination  Psychomotor hyperactivity | - | Lemborexant  Risperidone | 40 | 5 | 9 | Rejection of oral medication | Effective | Improvement of delirium | None | Improvement of delirium |
| 21 | Male | 58 | None | Use of steroid | Mixed delirium | Emotional excitement  Hallucination  Insomnia | Dexmedetomidine  Propofol | Lemborexant  Ramelteon | 80 | 12 | 12 | Inability to take oral medications | Effective | Remaining calm | None | Death |
| 22 | Male | 69 | None | Use of fentanyl | Mixed delirium | Emotional excitement | Prochlorperazine | Haloperidol | 20 | 3 | ≧14 | Expectation of stable medicinal effect | Effective | Improvement of delirium | None | Transfer to another hospital |
| 23 | Male | 79 | None | Cerebral infarction | Hyperactive delirium | Irritability  Violent behavior | Haloperidol  Lemborexant  Ramelteon  Yokukansan | Tiapride  Trazodone | 20 | Ineffective | 7 | Dangerous behavior | Ineffective | Persistence of delirium symptoms | None | Transfer to another hospital |
| 24 | Male | 72 | None | Diabetic ketoacidosis | Mixed delirium | Violent behavior  Psychomotor hyperactivity | - | Dexmedetomidine | 60 | 13 | 13 | Inability to take oral medication | Effective | Remaining calm | None | Improvement of delirium |
| 25 | Female | 81 | None | Inflammation of the bladder | Mixed delirium | Violent behavior  Irritability  Hallucination | Amantadine  Lemborexant  Levetiracetam  Trazodone | Risperidone | 20 | ≧14 | ≧14 | Insufficient efficacy with other drugs | Effective | Remaining calm | None | Improvement of delirium |
| 26 | Male | 78 | Yes | Use of steroid | Hyperactive delirium | Depression  Violent behavior | - | - | 20 | 8 | 8 | Inability to take oral medication | Effective | Remaining calm | None | Transfer to another hospital |
| 27 | Female | 85 | None | Chemotherapy | Mixed delirium | Hallucination  Psychomotor hyperactivity | - | - | 60 | 11 | 11 | Rejection of oral medication | Effective | Improvement of psychiatric symptoms | None | Transfer to another hospital |
| 28 | Male | 68 | None | Infection  (Pneumonia) | Hyperactive delirium | Psychomotor hyperactivity  Insomnia | - | - | 80 | 10 | 13 | Rejection of oral medication | Effective | Improvement of psychiatric symptoms | None | Improvement of delirium |
| 29 | Female | 87 | None | Heart failure | Mixed delirium | Psychomotor hyperactivity  Insomnia | - | Dexmedetomidine  Propofol | 20 | 5 | 7 | Insufficient efficacy with other drugs | Effective | Improvement of psychiatric symptoms | Somnolence | Improvement of delirium |
| 30 | Male | 70 | None | Post-surgery  (Arteriosclerosis obliterans) | Mixed delirium | Violent behavior  Psychomotor hyperactivity | - | Dexmedetomidine  Haloperidol  Lemborexant | 40 | ≧14 | ≧14 | Inability to take oral medication | Effective | Remaining calm | None | Transfer to another hospital |
| 31 | Male | 79 | None | Use of steroid | Hyperactive delirium | Emotional excitement  Violent behavior | Dexmedetomidine | Haloperidol  Midazolam | 60 | 7 | 12 | Insufficient efficacy with other drugs | Effective | Remaining calm | Somnolence | Improvement of delirium |
| 32 | Female | 86 | None | Hypernatremia | Mixed delirium | Insomnia  Irritability | - | Lemborexant  Ramelteon  Trazodone | 40 | 6 | ≧14 | Rejection of oral medication | Effective | Improvement of psychiatric symptoms | None | Transfer to another hospital |
| 33 | Male | 77 | None | Aortic dissection | Hyperactive delirium | Emotional excitement  Psychomotor hyperactivity | Dexmedetomidine | - | 80 | 7 | ≧14 | Rejection of oral medication | Effective | Remaining calm | None | Transfer to another hospital |
| 34 | Male | 76 | None | Heart failure | Mixed delirium | Hallucination  Insomnia  Violent behavior | Ramelteon  Suvorexant | Hydroxyzine pamoate | 40 | 5 | 13 | Insufficient efficacy with other drugs | Effective | Remaining calm | None | Improvement of delirium |
| 35 | Male | 72 | None | Hypercalcemia | Mixed delirium | Violent behavior  Hallucination | - | Chlorpromazine  Haloperidol  Hydroxyzine pamoate  Mirtazapine  Olanzapine  Trazodone  Quetiapine | 40 | 5 | 12 | Insufficient efficacy with other drugs | Effective | Remaining calm | Somnolence | Transfer to another hospital |
| 36 | Male | 81 | None | Heart failure | Mixed delirium | Hallucination  Insomnia | Dexmedetomidine  Propofol | Haloperidol  Lemborexant  Ramelteon | 60 | 3 | 9 | Avoidance of oral medication because of water restriction | Effective | Good sleep control | None | Improvement of delirium |
| 37 | Male | 80 | None | Pleural effusion | Hyperactive delirium | Irritability | Mirtazapine | Haloperidol  Hydroxyzine pamoate | 40 | Ineffective | 10 | Inability to oral medication | Ineffective | Persistence of delirium | Somnolence | Transfer to another hospital |
| 38 | Female | 83 | None | Distress due to breathing difficulties | Mixed delirium | Hallucination  Insomnia  Psychomotor hyperactivity | Brotizolam  Risperidone  Quetiapine | Dexmedetomidine | 20 | 6 | ≧14 | Insufficient efficacy with other drugs | Effective | Improvement of psychiatric symptoms | None | Transfer to another hospital |
| 39 | Male | 77 | None | Infection  (Pneumonia) | Mixed delirium | Hallucination | Haloperidol | - | 20 | 8 | ≧14 | Inability to oral medication | Effective | Improvement of psychiatric symptoms | None | Transfer to another hospital |
| 40 | Male | 92 | None | Heart failure | Mixed delirium | Hallucination  Insomnia  Psychomotor hyperactivity | - | - | 40 | 3 | 3 | Inability to oral medication | Effective | Improvement of psychiatric symptoms | None | Death |
| 41 | Male | 75 | None | Infection  (Pneumonia) | Mixed delirium | Emotional excitement  Hallucination  Insomnia | Etizolam | Dexmedetomidine | 40 | 9 | ≧14 | Inability to oral medication | Effective | Remaining calm | None | Improvement of delirium |
| 42 | Male | 85 | None | Use of hydromorphone | Mixed delirium | Hallucination  Psychomotor hyperactivity | Prochlorperazine | Olanzapine | 40 | 6 | 6 | Inability to oral medication | Effective | Improvement of psychiatric symptoms | None | Death |
| 43 | Male | 75 | Yes | Dehydration | Hypoactive delirium | Hallucination | Clonazepam  Perampanel | Haloperidol  Lemborexant  Perospirone | 80 | ≧14 | ≧14 | Dangerous behavior | Effective | Remaining calm | None | Discharge from hospital |
| 44 | Male | 80 | None | Heart failure | Hyperactive delirium | Violent behavior  Emotional excitement | Dexmedetomidine  Propofol | - | 80 | 3 | 5 | Inability to oral medication | Effective | Remaining calm | Dysphagia  (Extrapyramidal symptom) | Adverse event |
| 45 | Male | 83 | None | Infection  (Pneumonia) | Mixed delirium | Emotional excitement | Carbamazepine  Phenytoin  Phenobarbital | - | 20 | Ineffective | 5 | Inability to oral medication | Ineffective | Persistence of delirium | None | Death |
| 46 | Male | 74 | None | Heart failure | Hyperactive delirium | Psychomotor hyperactivity  Violent behavior | Ramelteon | Dexmedetomidine  Propofol | 20 | 11 | 11 | Rejection of oral medication | Effective | Remaining calm | Drooling  (Extrapyramidal symptom) | Adverse event |
| 47 | Female | 81 | None | Use of steroid | Hyperactive delirium | Confusion of thought  Emotional excitement  Psychomotor hyperactivity | Lemborexant  Ramelteon | Hydroxyzine pamoate | 40 | 3 | 4 | Rejection of oral medication | Effective | Remaining calm | None | Death |
| 48 | Female | 93 | None | Heart failure | Hyperactive delirium | Emotional excitement  Psychomotor hyperactivity | Clonazepam  Eszopiclone  Sertraline | Dexmedetomidine | 20 | 11 | 11 | Rejection of oral medication | Effective | Remaining calm | Drooling  (Extrapyramidal symptom) | Adverse event |
| 49 | Female | 72 | None | Use of fentanyl | Hyperactive delirium | Confusion of thought | Duloxetine  Zopiclone | Haloperidol  Hydroxyzine pamoate | 40 | Ineffective | 8 | Insufficient efficacy with other drugs | Ineffective | Persistence of delirium | Sedation | Transfer to another hospital |
| 50 | Male | 61 | None | Use of steroid | Hyperactive delirium | Confusion of thought  Emotional excitement  Irritability | Dexmedetomidine  Hydroxyzine pamoate  Propofol | Haloperidol  Mianserin  Suvorexant | 40 | Ineffective | 10 | Insufficient efficacy with other drugs | Ineffective | Persistence of delirium | None | Death |
| 51 | Male | 90 | Yes | Pneumonia | Hyperactive delirium | Violent behavior  Emotional excitement | Perospirone  Quetiapine  Ramelteon  Suvorexant  Yokukansan | Dexmedetomidine  Haloperidol | 40 | 3 | ≧14 | Insufficient efficacy with other drugs | Effective | Remaining calm | None | Transfer to another hospital |
